# The Role of a Local Health Department in Advancing Health Equity: Universal Postpartum Home Visiting in a Large Urban Setting

**DOI:** 10.1089/heq.2023.0104

**Published:** 2023-10-23

**Authors:** Kristine Zimmermann, Lisa S. Haen, Allissa Desloge, Arden Handler

**Affiliations:** ^1^ Department of Family and Community Medicine, University of Illinois College of Medicine Rockford, Rockford, Illinois, USA.; ^2^ Division of Community Health Sciences, School of Public Health, University of Illinois Chicago, Chicago, Illinois, USA.

**Keywords:** community collaboration, health equity, home visit, postpartum, universal

## Abstract

**Introduction:**

Racial and ethnic inequities persist among birthing families in urban U.S. communities, despite public health efforts to improve outcomes. To address these inequities, in 2020, the Chicago Department of Public Health (CDPH) launched Family Connects Chicago (FCC), an evidence-based, universal, postpartum home visiting program. We examine CDPH's transition from “high risk” to universal home visiting to determine whether and how this change represent an explicit commitment to advancing maternal and child health equity.

**Methods:**

We conducted a secondary analysis of key informant interview data (*n*=45 interviews) collected from stakeholders involved in FCC's early implementation. Our analysis involved identifying processes used by CDPH in their planning and early implementation of FCC and examining the alignment of these processes with approaches for promoting health equity proposed by Calancie et al.

**Results:**

The processes used by CDPH to plan and implement the FCC pilot are reflected in two major themes: (1) CDPH emphasized improving outcomes for all birthing families, and (2) CDPH prioritized engaging multiple stakeholders throughout planning and implementation. Alignment of these themes and their subthemes with the approaches proposed by Calancie et al. demonstrated that CDPH's implementation of FCC represents a commitment to advancing health equity.

**Discussion:**

In their planning and implementation of FCC, CDPH appears to have exhibited a concerted effort to address Chicago's persistent health inequities. Institutional commitment, continued stakeholder engagement, ongoing data sharing, and sustainable funding will be crucial to implementing and expanding FCC.

**Health Equity Implications:**

The implementation of FCC, a new service delivery approach for maternal and infant health, marks a new beginning in tackling inequities for Chicago's birthing families.

## Introduction

Birthing families in cities across the United States experience persistent racial and ethnic inequities in maternal and child health (MCH) outcomes. For example, in Chicago in 2020, the mortality rate for Black infants was over five times as high as that for White infants (10.5 and 2.0 deaths per 1000 live births, respectively), and the rate for Hispanic infants was over twice as high (4.1 deaths per 1000 live births).^1^ Similarly, from 2011 to 2016, Chicago's pregnancy-associated mortality ratio was almost six times higher for non-Hispanic Black women (98.8 per 100,000 births) and two times higher for Hispanic women (34.3 per 100,000 births) compared to non-Hispanic White women (17.0 per 100,000 births).^2^

These inequities are increasingly attributed to issues of structural racism, including residential segregation, which can impede access to high-quality reproductive health care and expose families of color to stressors such as unsafe neighborhoods and environmental hazards.^3–6^ Despite public health efforts, racial inequities in MCH outcomes persist.^7,8^ Furthermore, while MCH policy-makers and advocates have provided a clear rationale for addressing inequities, guidance on how to do so is limited.^9^

In Chicago, birthing families have historically been supported by a complex system serving those deemed “at risk.” Organizations, including the Chicago Department of Public Health (CDPH), have offered programs with varying objectives and eligibility criteria, resulting in some families being offered multiple, often duplicative programs, while others are not reached at all. In response to the fragmented service delivery system and persistent MCH inequities, CDPH sought an alternative approach. In 2020, CDPH launched Family Connects Chicago (FCC), based on the Family Connects home visiting model.^10–14^ Family Connects offers a postpartum nurse home visit to all families at around 3 weeks postpartum. The visit includes a comprehensive family need assessment, education, and referrals to services, including intensive home visiting, if appropriate.

**FIG. 1.**
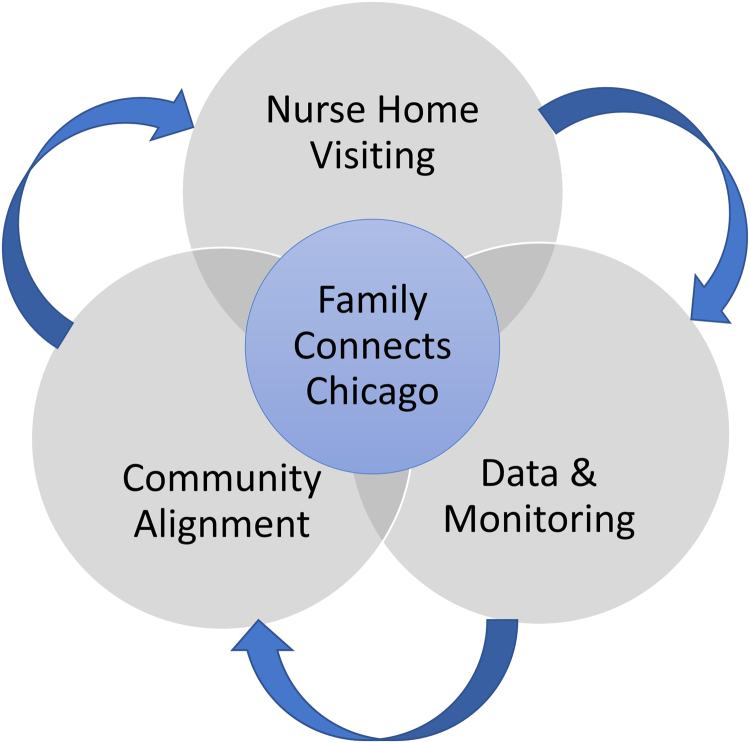
Family Connects Chicago Model. FCC integrates postpartum nurse home visiting, data collection and monitoring, and a community alignment process to support services to families and systems-level change. FCC, Family Connects Chicago.

Family Connects also involves a “community alignment process” in which individual family needs data are used to identify and address resource gaps. Thus, Family Connects is designed to improve family outcomes and address persistent service delivery gaps through systems change (Fig. 1). The Family Connects model initially focused on improving population-level pediatric outcomes, including reduced emergency care visits.^11^ More recently, the Family Connects model has been acknowledged as a health equity approach due to its use of a comprehensive family risk assessment to assess acute health needs as well as family needs related to health care access, parenting, household safety, and parental well-being; the use of family needs data to improve community systems of care; and effectively connecting families to community resources to address their needs.^14–17^

From 2020 to 2021, CDPH led the FCC pilot implementation in collaboration with four hospitals, three “Regional Community Alignment Boards” (RCABs), and a Citywide Advisory Council. The Family Connects model typically uses a single community advisory board; however, due to its size, Chicago was divided into six regions, with an RCAB serving each region, and the Citywide Advisory Council provided high-level oversight. During the pilot, FCC nurse home visitors were employed by CDPH and one partner hospital. A team of researchers conducted an external evaluation of the FCC pilot to assess early implementation barriers and facilitators, identify best practices before citywide expansion, and inform a future impact evaluation.^18^

According to CDPH, FCC was an explicit effort to address the persistent maternal and infant inequities in Chicago,^19,20^ including many of the most severe outcomes—which manifest in the postpartum period and are preventable—including maternal death and infant death due to Sudden Unexpected Infant Death/Sudden Infant Death Syndrome.^5,6^ This secondary analysis of the qualitative data collected for the FCC pilot evaluation examines whether and how CDPH's commitment to a health equity approach was demonstrated during the FCC pilot implementation. As the first large urban local health department to implement the Family Connects model, CDPH's experience approaching health equity through universal home visiting can inform similar efforts in other urban settings.

## Methods

Between January 2020 and June 2022, a team of researchers from the University of Illinois Chicago (UIC) School of Public Health conducted the external evaluation of the FCC pilot with funding from four private foundations; funding for the evaluation was independent from the funding of the pilot itself. The FCC pilot evaluation involved a multiple-methods approach, including qualitative key informant interviews to understand the experiences and perspectives of stakeholders involved in FCC's early implementation.

### Study design

Interviews were conducted between April 2020 and February 2022 with CDPH leaders involved in FCC, nurse home visitors from CDPH and a partner hospital, clinical and administrative personnel from three partner hospitals, organizational staff and partners from RCAB delegate agencies, and Citywide Advisory Council members. This study used a health equity lens to conduct a secondary analysis of these interview data. The study was approved by the UIC and CDPH Institutional Review Boards.

### Key informant interviews

#### Identification and recruitment

The researchers identified and selected potential interviewees using both purposive and convenience sampling to elicit a range of perspectives. Specifically, the researchers used lists of individuals involved in implementation to prioritize a heterogeneous sample of participants based on characteristics such as their implementation role and organization and demographic characteristics, and for CDPH nurse home visitors, length of time in their current role. Researchers contacted potential interviewees to schedule interviews and completed interviews with all who agreed to participate.

#### Key informant interview implementation

The research team used the evaluation objectives, a previous Family Connects implementation evaluation,^21^ and implementation science concepts^22,23^ to develop a semistructured interview guide focused on interviewee experiences with and perceptions about FCC's implementation, expansion throughout Chicago, and potential for improving Chicago's MCH service delivery system. Three researchers (A.D., L.S.H., and K.Z.) conducted one-on-one interviews by phone or Zoom (Zoom Video Communications, Inc., San Jose, CA). Interviews were audio-recorded and transcribed verbatim. In total, 35 key informants participated in one interview, and five participated in two interviews each (in pilot years 1 and 2) to capture early implementation perspectives and lessons learned (Table 1).

**Table 1.** Family Connects Chicago Key Informant Interview Participants (*n*=40)

	***n*** (%)
Interviewee role	
Nurse home visitor	13 (32.5)
CDPH personnel	4 (10.0)
Hospital personnel	9 (22.5)
RCAB delegate agency staff	5 (12.5)
RCAB partner	5 (12.5)
Citywide advisory council member	4 (10.0)
Race/Ethnicity	
Asian	2 (5.0)
Black/African American	17 (42.5)
Hispanic/Latinx	8 (20.0)
White	13 (32.5)
Education	
Bachelors or less	11 (27.5)
Masters	21 (52.5)
Doctorate (e.g., MD, PhD)	8 (20.0)
Interview year	
Year 1 only	10 (25.0)
Year 2 only	25 (62.5)
Years 1 and 2	5 (12.5)

CDPH, Chicago Department of Public Health; RCAB, Regional Community Alignment Board.

### Data analysis

For the pilot evaluation, the researchers analyzed interview data using a deductive qualitative content analysis approach.^24^ Two researchers (L.S.H. and K.Z.) developed a codebook based on the evaluation's objectives and implementation science concepts.^22,23,25^ They used an iterative process with a cross-section of transcripts from all stakeholder types to create a final codebook consisting of 30 codes.

Each transcript was coded by at least two of three researchers (A.D., L.S.H., and K.Z.) to ensure coding agreement. The team discussed discrepancies until consensus was reached. The team met weekly to discuss progress, coding discrepancies, and emerging findings. The senior member of the team (A.H.) also assisted with the interpretation of findings. Analysis was facilitated using Dedoose qualitative data analysis software (Dedoose Version 9.0.46, Los Angeles, CA: SocioCultural Research Consultants, LLC, www.dedoose.com).

Understanding CDPH's commitment to health equity was not the primary focus of the FCC pilot evaluation, which centered on practice-focused implementation and implications for expansion. However, given CDPH's stated commitment to health equity,^19,20^ in this analysis, the team explored whether this expressed commitment to health equity was demonstrated in the qualitative data. To do so, the team identified the processes used by CDPH to select and implement FCC, and over the course of multiple meetings, organized the data into categories, examined the categories for patterns, used an iterative process to synthesize data patterns into themes and subthemes, and then explored whether these themes aligned with the health equity approaches proposed by Calancie et al.^9^ (Table 2).

**Table 2.** Approaches for Promoting Health Equity in Communities, Adapted from Calancie et al.

Approach	Description
A	Expand the understanding of the drivers of health and work across sectors: Acknowledging that the structural and social determinants of health are the major drivers of health status. Furthermore, these determinants are complex and interconnected, requiring multisectoral collaborations to make changes in the pursuit of health equity.
B	Take a systems approach: Understanding how and why systems advantage some individuals and disadvantage others can be examined through systems thinking. Systems thinking can help identify multifactorial problems, bust silos, and uncover cross-sector opportunities for policy change.
C	Reflect on your own organization: Looking inward to examine how diversity, equity, and inclusion manifest within one's own organization can influence the perspectives brought to community-based efforts.
D	Follow the lead of communities who experience injustices: Every step of developing strategies to promote health equity, from determining needs to interpreting data, should center the knowledge and perspectives brought forth by community members.
E	Work with community members, decision-makers, and other stakeholders to prioritize action: It is essential to align health equity solutions with community-identified strengths, needs, priorities, and constraints. In doing so, flexibility and adaptability are critical, so that changes can be made based on stakeholder input.
F	Foster agency within individuals and collective action within groups: In efforts to address inequities, those with power need to direct support and financial resources to those with lived experience. Sharing power develops agency in individuals and fosters collective action within groups.
G	Identify and collect data to show where health inequities currently exist to inform equitable investment of resources: In taking a community-engaged approach to implementing interventions, it is important to work with communities to identify data sources, determine outcomes of interest, and analyze and interpret the data in a way that is credible and does not promote false narratives or biases.
H	Be accountable to outcomes that reflect real improvements in people's lives: Organizations need to think critically about their goals and objectives and align their resources with an outcome-oriented approach. Asking questions that engage individuals and communities is key to addressing inequities.

## Results

We identified two main themes with associated subthemes, which reflected the processes used by CDPH in selecting and implementing the FCC pilot: (1) Commitment to improving outcomes for all birthing families and (2) engagement of a broad range of stakeholders in FCC planning and implementation. Below we provide descriptions of these themes and their subthemes and indicate their alignment with Calancie et al.'s recommended approaches.^9^ Making explicit the relationship between CDPH's processes and the Calancie approaches to health equity illustrates how CDPH's selection and implementation of FCC demonstrate a commitment to health equity.

### Theme #1: CDPH emphasized improving outcomes for all birthing families

In identifying the need to change its MCH service model and throughout the planning and implementation of FCC, CDPH recognized that its High Risk Infant Follow-up (HRIF) program, a public health nurse home visiting program providing long-term follow-up for infants identified as high risk at birth, was only reaching a fraction of Chicago's families. Furthermore, despite consistent efforts, stark inequities in access to resources and health outcomes for Chicago's birthing families remained, with Black families living on Chicago's South and West sides bearing a disproportionate burden of these inequities. CDPH was determined to *be accountable to outcomes affecting people's lives (Approach H)* by reaching more families, including high-risk families not served by HRIF.

#### Subtheme #1a: CDPH recognized HRIF was not having the desired population-level effect

Before selecting the Family Connects model, CDPH acknowledged that Chicago's persistent MCH inequities had not changed over decades of implementing its HRIF program.

*Our nurses have been doing some kind of home visit work for decades… The city was not seeing the changes we wanted to see in infant and maternal mortality. If anything, it's increased, especially for Black and Brown communities*. (CDPH, Year 1)

This recognition of persistent MCH inequities and the need to change course demonstrate CDPH's commitment to *understanding the drivers of health* and *being accountable to outcomes affecting people's lives (Approaches A, H)*.^9^

#### Subtheme #1b: CDPH and stakeholders selected an evidence-based, “universal” home visiting approach

To address these inequities, and due to the lack of evidence for the HRIF program, CDPH engaged multiple stakeholders to select a new service model.

*The service that we were offering prior to [FCC], there was no evidence, science behind the effectiveness and outcomes of what we were delivering.* (CDPH, Year 2)

The stakeholders selected the evidence-based Family Connects model,^26^ acknowledging that, while evidence-based interventions do not necessarily work for all populations in all communities, compared to the HRIF program, the Family Connects model offered promise as a way to *improve outcomes affecting people's lives (Approach H)*. In addition, as a universal approach designed to serve all families, FCC had the potential to achieve a greater reach across Chicago. While the FCC pilot was implemented in only four hospitals, it was universally available to all birthing families in these hospitals with the long-term goal of expanding to all Chicago hospitals. Furthermore, CDPH intentionally sought to collaborate with hospitals for the pilot in some of the city's most under-resourced communities to ensure the pilot was feasible and effective in these communities before expanding across Chicago.

*When you're looking through like an equity lens…we look at data around our communities… Are we servicing people in…what we consider “higher risk” community? If so, what are we seeing about those families, and are we getting them connected to services? Are they getting what they need?* (CDPH, Year 2)

By selecting an evidence-based, universal approach, and prioritizing pilot implementation in communities experiencing the greatest risks, CDPH led a holistic health equity approach. Selecting a universal approach aligns with CDPH's commitment to *following the lead of communities experiencing injustices* and being *accountable to outcomes affecting people's lives (Approaches D, H),*^9^ and selecting the highest-risk communities for pilot implementation reflects a commitment to *targeting resources to the areas where needs are the greatest (Approach G).*

#### Subtheme #1c: CDPH and stakeholders selected the Family Connects model as an approach to address systems-level drivers of inequities

CDPH and its stakeholders recognized that fragmented systems of care for Chicago's birthing families contributed to inequitable distribution of services and resources, and lack of coordinated services for birthing families resulted in duplicative, competing, or missing services.

*There's also pockets of the city where there's a lot of services and people are getting three different home visit programs. And then, there's pockets of the city where folks aren't getting any of those types of services… There's not equity…in terms of what's available… The systems don't talk to one another.* (CDPH, Year 1)

Thus, CDPH and stakeholders sought to reduce inequities by selecting a model that would foster collaboration among health and social service providers across Chicago. In particular, FCC's “community alignment” component is designed such that family needs identified during home visits are aggregated to reveal resource gaps to be shared with and addressed by the RCABs or Citywide Advisory Council. For example, using family data collected during the pilot, CDPH identified and prioritized areas of need that were addressed with short-term resources (e.g., distribution of diapers and cribs), as well as long-term resource and policy change needs.

*Families were reporting challenges with acquiring diapers and diaper wipes and pack-n-plays…those really essential items… These additional dollars we were able to get to our [RCAB] delegates that they could purchase those items…that was a really early win in terms of being able to quickly see from the data what's an intervention that might be helpful and then be able to actually move on it.* (CDPH, Year 2)

While short-term needs were addressed during the pilot, interview participants recognized that systems-level changes, such as access to resources to reduce housing instability, would take more time. Interview participants perceived the community alignment structure as a promising mechanism to address these gaps.

The Family Connects model is structured such that programmatic data are intended to elucidate changes needed at the systems level. Thus, CDPH's selection of FCC also demonstrates their commitment to *using data to understand drivers of inequities, using a systems approach to address these gaps, and identifying and collecting data to inform investment of resources (Approaches A, B, G).*

*As we get more into our data…we're looking at how we're doing in communities, how we're reaching folks, where we have gaps… There's going to be things we need to address at a community level… But there are going to be things that we're truly talking about system change, that are going to require some policy change… Maybe a change in the way we think about funding…how we allocate dollars to communities*. (CDPH, Year 2)

#### Subtheme #1d: CDPH prioritized evaluation and data sharing with FCC program stakeholders

To ensure improved outcomes for birthing families remained a focus in FCC, CDPH prioritized the collection, use, and sharing of data and data-driven decision-making. For example, CDPH developed data dashboards for stakeholders to access FCC data and *provide input for program improvement and action (Approach E)*. Data collected during the pilot evaluation were also used to *inform FCC's city-wide expansion (Approaches G, H).*

*At the last meeting, it was so great…to be able to really start seeing those numbers come out, so we're really seeing solid data, being able to hear stories about what impact that this is having on the families… Being able to see the data, I also think it's uncovering things that need to be examined in order to really roll this out more broadly..*. (Citywide Advisory Council Member, Year 2)

### Theme #2: CDPH prioritized engaging multiple stakeholders throughout FCC planning and implementation

To achieve the goal of improved outcomes, CDPH recognized the need for engaging partners at all levels, including community members, organizations, and health systems. As such, CDPH leadership consistently engaged stakeholders in the planning and implementation of FCC and planning for FCC expansion, extending beyond “advisory” roles *(Approach E). Community stakeholders were often co-leaders* in the FCC pilot *(Approach F),* involved in prioritizing and initiating action to address community needs.

*Our intention was to have as many stakeholders that we know are involved with providing a strong beginning for families, and making sure we're getting folks who have that expertise…who have been involved in MCH for a while or in the community… They know and they will be able to tell us whether the implementation or the program itself is working or will work for their communities and the needs that they have*. (RCAB Staff, Year 2)

#### Subtheme #2a: Before implementation, CDPH engaged community stakeholders to understand Chicago's MCH landscape

In 2018, CDPH began their process to understand and address the root causes of MCH inequities and explore alternative strategies for reaching birthing families. This involved *engaging multiple stakeholders* from *communities experiencing injustices* to *understand the drivers of health (Approaches A, D, E),* to fully understand the MCH landscape in Chicago.

*I think we had more than 160 people participate in a series of regional roundtables…in the information gathering phase that we were asking this question, “Here's the program we were doing. We don't have outcome data from that program. [And] we never turned around maternal, and the infant mortality, morbidity are going in the wrong direction. So, what should we be doing differently?”* (CDPH, Year 2)

Stakeholders recognized that, while families deemed “high risk” were receiving support through the HRIF program, this support was not addressing systemic and structural inequities for all birthing families, including systemic racism.

*These types of services aren't always needed just by those that have low socioeconomic income… For example, infant and maternal mortality, that is a race-based conversation, whether you're upper income or lower income.* (Citywide Advisory Council Member, Year 2)

#### Subtheme #2b: CDPH continued to engage multiple stakeholders and use a collaborative approach in the FCC pilot implementation

After selecting FCC, CDPH continued to *prioritize involvement by stakeholders, including community members, community-based organizations, and health systems (Approach E)*. CDPH developed the Citywide Advisory Council, which provided high-level oversight; an RCAB structure to ensure community-level engagement; and later, a Health Care Providers Council to integrate the clinical care delivery system.

*I think it's important that we're continuing to work together to fill those gaps because nobody can do it on their own, but if we're pooling the resources of the City of Chicago and different hospitals in the area and different nonprofits. I think it's so important…getting those different stakeholders involved in working together.* (Hospital Administrator, Year 1)

#### Subtheme #2c: RCABs were developed as empowered entities to systems change

A key component of the Family Connects model is community alignment, in which community stakeholders identify and take action to address community needs. In the FCC pilot, the RCABs were implemented by three community-based organizations, which received support and funding from CDPH, but operated independently. One role of the RCABs was to review FCC data to *identify and implement potential system responses (Approaches B, G*). In addition, by using a regional structure, the RCABs could focus on *community-specific needs and priorities, while also considering community assets and strengths (Approach E).*

*We're thinking about establishing subcommittees to work on some of the priorities that the CAB members identified… By having the subcommittees and folks being able to opt into particular topics and issues that they're most interested in, I think that also we'll be able to provide other opportunities for folks to work with one another on specific issues, cross collaborate, to support the Family Connects.* (RCAB Staff, Year 2)

#### Subtheme #2d: CDPH provided support and training for culture change among their nurse home visitor workforce

In shifting to FCC from the HRIF program, CDPH initially faced resistance from nurse home visitors, who were accustomed to establishing long-term relationships with families and were concerned FCC might be inadequate for Chicago's most vulnerable and marginalized families. In time, however, CDPH nurse home visitors recognized their increased autonomy within FCC for engaging with families, their critical roles in educating families and other nurse home visitors newly hired for FCC about community resources, and their opportunities to raise awareness about service gaps with RCABs. Thus, the *nurse home visitor role was transformed beyond care provider to agent of change (Approaches E, F)*.

*If there is [a resource for families] that's missing, you bring it up at the case conference… They take it back to their [RCAB] meetings and they talk about it and…how they could get funding for it, how they can either locate somewhere that they can do it… And the great part is that the nurses with their ideas—like I had two ideas and other nurses had ideas, and they can use those ideas. Whereas before, they never would have heard my idea.* (Nurse Home Visitor, Year 2)

## Discussion

The World Health Organization recommends postpartum home visits for all women and infants.^27^ Universal MCH services, including home visiting, common in Western Europe, are thought to partially explain their historically better outcomes.^28^ Benefits of universal home visiting can include reduced stigma associated with receiving services, increased program participation, and increased community and political support for programming due to the broader reach.^10^

In the United States, postpartum home visiting is typically neither routine nor universal. Furthermore, despite numerous public health programs targeting birthing families, systemic racism, and other injustices facing urban communities are reflected in persistent inequities in MCH outcomes. To address these inequities in Chicago, CDPH engaged in a major structural shift from implementing a long-term High Risk Infant Follow-up program serving 1–3% of birthing families between 2013 and 2019, to FCC, a short-term, postpartum, nurse home visiting program for all families.^8,11,24,25,27^

Using the health equity approaches outlined by Calancie et al. as a framework,^9^ we provide evidence of how CDPH's effort to transform MCH services in Chicago demonstrates an explicit effort to advance health equity. Specifically, for CDPH, the opportunity to engage MCH stakeholders to collaboratively implement an evidence-based program aimed at all families, while targeting the most vulnerable, and prioritizing evaluation of the FCC pilot to inform citywide expansion, were critical factors in its selection of FCC and represent distinct, but interrelated health equity approaches.

Calancie et al. suggest that the health equity approaches they identified are not a “recipe,” but concepts that can be customized for specific communities.^9^ Our evidence suggests these concepts often overlap, particularly the importance of collaboration with stakeholders and the elevation of communities most affected by injustices. CDPH leaders recognized they could not plan and implement FCC without engagement of a wide range of MCH stakeholders and professionals, including neighborhood representatives and community organizations, hospitals and health care workers, and philanthropic and governmental stakeholders.

Beyond stakeholder engagement, CDPH also recognized the need for shared leadership between the health department and many others across Chicago's MCH landscape. Importantly, organizational partners within vulnerable communities are often best positioned to address community needs, when they have adequate capacity and resources to do so.^29^ As such, in the FCC pilot, the RCABs did not merely serve in an advisory role; they were provided with autonomy over their structure and function and were provided funding to accomplish their goals. As funders and governmental agencies increasingly require collaboration for systems change,^30,31^ community stakeholders must not only be included as collaborators but also need to be elevated as leaders and provided with resources to ensure their capacity to contribute to equity and related initiatives.^29^

Calancie et al. also identify the critical role of data to understand health inequities, inform investment of resources, and ensure accountability.^9^ In the FCC pilot, CDPH emphasized the importance of routine data collection and data sharing to support ongoing program implementation and inform systems change.

In summary, our secondary analysis demonstrates that CDPH's selection and implementation of FCC reflect its commitment to using a health equity approach to MCH programming and resource distribution. However, we also posit that CDPH's action to advance health equity by introducing a universal model, while targeting their initial efforts to the most vulnerable communities, is atypical for a large urban health department, given the challenges with adopting significant programmatic and cultural changes within sizable, bureaucratic organizations. First and foremost, major programmatic change by a large urban health department is not easily achieved without the concomitant institutional commitment from the highest levels of local government. In the case of CDPH, it is evident that FCC directly aligns with broader initiatives within the department (e.g., Healthy Chicago 2.0, Healthy Chicago 2025), which have involved communities in designing responses to health inequities with a focus on structural and social determinants of health.^32,33^

However, even with internal support, an undertaking of this nature might not be easily replicated. Funding for both the HRIF program and the FCC pilot was mainly derived from a mini-block grant provided by the Illinois' MCH/Title V program. FCC's expansion is currently being bolstered by funding from the American Rescue Plan Act. Therefore, sustaining FCC in Chicago and expanding to other large cities will require a viable long-term funding mechanism. Allowing Medicaid and insurance reimbursement for universal home visiting, while providing infrastructure support from city and state funding sources, would help to sustain and grow the program in Chicago and other locales.

As an evidence-based model that includes a process for engaging in systems change, it is anticipated and hoped that FCC will be more effective in improving and aligning the MCH service delivery system in Chicago than past efforts. Ultimately, overall decreases in maternal and infant morbidity and mortality and reductions in the vast inequities between White birthing persons and Black and other persons of color in Chicago will be the true test of this approach.

This study has several limitations. Although the evaluation team collected a robust set of qualitative data, these data did not explicitly focus on the role of health equity in CDPH's adoption and implementation of FCC. As such, the alignment between the CDPH processes and health equity approaches promulgated by Calancie et al.^9^ was conducted in a *post hoc* manner. In addition, due to the research team's positionality as public health researchers in Chicago analyzing interviews from stakeholders who were largely enthusiastic about FCC, our interpretation of findings may be biased by the promise offered by a novel approach to services.

Furthermore, despite what appears to be CDPH's clear commitment to health equity, the effects of FCC on addressing structural drivers of inequities and MCH outcomes will not be known for several years. Finally, FCC was initiated simultaneous with the coronavirus disease 2019 (COVID-19) pandemic. Since we have evidence that maternal mortality, for example, increased during COVID-19,^34^ positive improvements in MCH outcomes may take longer to manifest than might have been the case without COVID-19. Despite these limitations, we believe the experience of CDPH in launching FCC analyzed through a health equity lens is informative in describing how a large health department can undertake a major change in its MCH programming to support and invest in a health equity approach.

### Health equity implications

Successful approaches to achieving health equity include institutional commitment, collaboration, shared leadership across key stakeholder groups, and prioritizing data collection and sharing. Likewise, selection and implementation of an intervention grounded in equity can lead to “real improvements in people's lives.” This is the promise of the Family Connects model, which recognizes the early postpartum period as an ideal time to provide a universal touch for all families and incorporates strategies to move from individual needs to systems change. As such, the launch of FCC in Chicago in 2020 marks a new beginning in the ability of Chicago's MCH service delivery system to truly meet the needs of birthing families and tackle persistent inequities in maternal and infant outcomes.

## Abbreviations Used

CDPH: Chicago Department of Public Health
COVID-19: coronavirus disease 2019
FCC: Family Connects Chicago
HRIF: High Risk Infant Follow-up
MCH: maternal and child health
RCAB: Regional Community Alignment Board
UIC: University of Illinois Chicago
